# 

**Published:** 1914-07-11

**Authors:** 


### CONTENTS.

| | PAGE |
|---|---|
| **A Great City's Hospital Work** ... ... ... | 397 |
| **National Insurance and the Poorer Hospitals**... | 398 |
| **Hospital and Institutional News** ... ... ... | 399 |

Their Majesties at Glasgow Royal Infirmary—At the Royal Hospital for Sick Children—The Consumption Conference at Leeds—Surgical Tuberculosis in Childhood—The Royal Salop Infirmary—The New Sanatorium at Hull—Hospital Expansion in Birkenhead—The Birmingham Concerts Committee—The Royal Albert Infirmary, Wigan—A Leper Hospital in Essex—More Medical Archæology—The New Appointments at St. George's Hospital—The Secretaryship of St. Mark's Hospital, City Road—The Devonshire Hospital at Buxton—The Bristol General Hospital—Proposed Cottage Hospital at Maltby—Undergraduates and Voluntary Hospitals—An Institutional Pilgrimage—Sanatoria and After-care—Colossal Endowments in America—A Flourishing Concern—Judge Mellor on a Bad Practice—The Council Election of the Royal College of Surgeons—Nightingale Scholarships—The War Office Committee on Voluntary Aid Detachments—Deaths by Poisoning—This Week's Drug Market.

| | PAGE |
|---|---|
| **Modern Methods Series** ... ... ... ... | 405 |
| The Nascent Iodine Treatment of Tuberculosis. | |
| **British versus Continental Spas** ... ... ... | 406 |
| A Plea for Home-made Hydrotherapy. | |
| **Emetine in the Dysentery of Children** ... | 406 |

[*See over.*]

### CONTENTS—(*continued*).

PAGE
**Reports on Hospitals of the United Kingdom**— 407
By SIR HENRY BURDETT, K.C.B., K.C.V.O.
The North Evington Infirmary, Leicester: Report of the Guardians' Committee on Sir Henry Burdett's Report.
**Buildings Contemplated and in Progress** ... 409
**Relations of Hospitals with Panel Practitioners** 410
The London Panel Committee and the Budget Grants.
**Institutional Organisations in America** ... 411
I. Some Reforms in Children's Out-patient Clinics.
**The Coroners' Society** ... ... ... ... 412
Dr. F. J. Waldo on Imperial and Local Government.
**National Insurance Act Developments** ... ... 413
The Friendly Societies' Conferences.
Insurance Act Visitors.
**The Institutional Library** ... ... ... ... 415
A Medical Classic—A Series from the Hospital Necker.

PAGE
**The Glasgow Royal Infirmary Chairman** ... 416
**Round the World** ... ... ... ... ... ... 417
Fellow-Workers and their Doings.
**The Department of Modern Nursing** ... ... 418
IV. West London Hospital.
**The Editor's Letter-Box** ... ... ... ... 419
Royal Hospital for Diseases of the Chest—The Colour Problem in Hospital Life—Hospital Plans and their Selection—Graceful Giving.
**The Annual Meeting of the Poor-Law Medical Officers' Association of England and Wales** 420
**The Profession of Medicine** ... ... ... 421
Medical Representatives on Insurance Committees.
The British Medical Association in Scotland.
Strike of German Doctors.
**The National Medical Union (Official)** ... ... 422
Proceedings at General Meeting.
**Hospital Equipment** ... ... ... ... ... 424
**The Institutional Worker** ... ... (Suppl.) 1
Destructors for the Disposal of Hospital Refuse.
Collar Ironing Machines.

